# Infrared photodetector sensitized by InAs quantum dots embedded near an Al_0.3_Ga_0.7_As/GaAs heterointerface

**DOI:** 10.1038/s41598-020-68461-w

**Published:** 2020-07-15

**Authors:** Takahiko Murata, Shigeo Asahi, Stefano Sanguinetti, Takashi Kita

**Affiliations:** 1 Department of Electrical and Electronic Engineering, Graduate School of Engineering, Kobe University, 1-1 Rokkodai, Nada, Kobe, 657-8501 Japan; 2 L-NESS and Dipartimento di Scienza dei Materiali, Università di Milano-Bicocca, 20125 Milan, Italy

**Keywords:** Optical sensors, Quantum dots

## Abstract

Mid-infrared sensors detect infrared radiation emitted from objects, and are actually widely used for monitoring gases and moisture as well as for imaging objects at or above room temperature. Infrared photodetectors offer fast detection, but many devices cannot provide high responsivity at room temperature. Here we demonstrate infrared sensing with high responsivity at room temperature. The central part of our device is an Al_0.3_Ga_0.7_As/GaAs heterostructure containing InAs quantum-dot (QD) layer with a 10-nm-thick GaAs spacer. In this device, the electrons that have been accumulated at the heterointerface are transferred to the conduction band of the Al_0.3_Ga_0.7_As barrier by absorbing infrared photons and the following drift due to the electric field at the interface. These intraband transitions at the heterointerface are sensitized by the QDs, suggesting that the presence of the QDs increases the strength of the intraband transition near the heterointerface. The room-temperature responsivity spectrum exhibits several peaks in the mid-infrared wavelength region, corresponding to transitions from the InAs QD and wetting layer states as well as the transition from the quantized state of the triangular potential well at the two-dimensional heterointerface. We find that the responsivity is almost independent of the temperature and the maximum value at 295 K is 0.8 A/W at ~ 6.6 µm for a bias of 1 V, where the specific detectivity is $1.8\times {10}^{10}$ cmHz^1/2^/W.

## Introduction

Various physical mechanisms allow us to probe thermal radiation, and accordingly various different device designs for infrared (IR) detectors have been proposed^1–6^. These sensors detect mid-infrared radiation emitted from objects, and are actually widely used for monitoring gases and moisture as well as for imaging objects at or above room temperature^3,5,7^. IR detectors can be classified into thermal detectors and photodetectors. Generally, thermal detectors have been widely used for IR detection as they have certain advantages^4,8^: their responsivity is almost independent of the wavelength and they can operate at room temperature. However, the response of a thermal detector is very slow and the sensitivity is usually lower than that of a photodetector. Conversely, photodetectors utilizing interband transitions to absorb IR photons have important practical advantages such as fast response, high sensitivity, and wavelength selectivity (for example, HgCdTe^9–11^ is able to cover a wide wavelength range from 1 to 25 µm by controlling the Hg content^11^). However, due to the narrow band gap required for IR-induced interband transitions, these photodetectors are easily affected by thermal noise, and thus require low operating temperature (less than ~ 100 K) to achieve high signal-to-noise ratios. To reduce the dark current and improve the sensitivity, photodetectors using quantum nanostructures such as quantum wells (QWs)^12–22^, quantum dots (QDs)^23–39^, and dots in a well (DWELL)^40–46^ of semiconductors with relatively wide band gaps which can suppress intrinsic carrier generation have been developed. Devices based on QWs are called QW IR photodetector (QWIP) and those based on QDs (including DWELL) are the so-called QD IR photodetectors (QDIPs). For sensing, these devices utilize *intraband* (intersubband) optical transitions to absorb the IR light. Since intraband transitions in QWs are forbidden for IR light normally incident on a QW slab (parallel to the sensor surface) according to the optical selection rule, QDs are more suited for realizing normal-incidence IR sensors. In particular, the application of InAs/GaAs QDs to QDIPs has been intensively studied^47–54^, because of the well-established growth of self-assembled QDs on GaAs substrates. The reported responsivity range of QDIPs enables detection of mid-wavelength IR (MWIR) light at 3 to 5 µm and long-wavelength IR (LWIR) light at 8 to 12 µm. However, the required operation temperature is still too low. Photodetectors that can be used at room temperature have been reported^3,10,18–20,26,31,38,39^, but the responsivity is relatively low if the device operates at room temperature. An improved device design for room-temperature operation would be beneficial for various applications.

The operating principle of a photodetector resembles that of a solar cell, and thus, it seems feasible to redesign a suited solar cell in order to realize IR sensing at room temperature. Recently, we proposed and demonstrated the so-called two-step photon up-conversion solar cell (TPU-SC)^55–58^. This device basically consists of a single heterointerface between an intrinsic Al_0.3_Ga_0.7_As layer and an intrinsic GaAs layer, embedded between *n*-doped Al_0.3_Ga_0.7_As and *p*-doped GaAs layers to form a *p*–*i*–*n* junction. The photons with energy below the band gap energy of Al_0.3_Ga_0.7_As but above that of GaAs, can directly excite the latter semiconductor and thus generate electrons near the heterointerface. The electrons that have been excited in the *i*-region of GaAs, drift towards the heterointerface due to the internal electric field and accumulate at the heterointerface. Finally, they are pumped into the Al_0.3_Ga_0.7_As barrier by absorbing IR photons with energy below the band gap energy of GaAs. This final step is accomplished by the intraband transition at the heterointerface, and we found that efficient intraband transition can be achieved by introducing InAs QDs near the heterointerface. Generally, intraband transitions at a two-dimensional heterointerface are forbidden for light with the polarization components parallel to the heterointerface. Conversely, three-dimensionally confined QD allows intraband transitions for all the polarization directions. When QDs are inserted near the two-dimensional heterointerface, InAs QD breaks the intraband optical selection rule and, therefore, acts as a sensitizer for IR light shining on the device. Therefore, the important key structure realizing the excellent IR detection feature at room temperature is the combination of the heterointerface and quantum dots. The heterointerface enables to highly accumulates electrons, and QDs improve the sensing in the mid IR wavelength region. The initial state with the high-density electrons for the intraband transition is the great advantage against conventional devices using QDs called QDIP in which the density of states occupied with electrons is low as compared to ours because of a limited quantum dot density. This design principle should be useful for realizing highly sensitive IR absorption at room temperature. In this work, we develop *n–i–n* photodetectors sensitized by InAs QDs embedded near an Al_0.3_Ga_0.7_As/GaAs heterointerface and demonstrate a high IR responsivity and a specific detectivity at 295 K.

## Results

### Concept of our device structure

**Figure 1:**
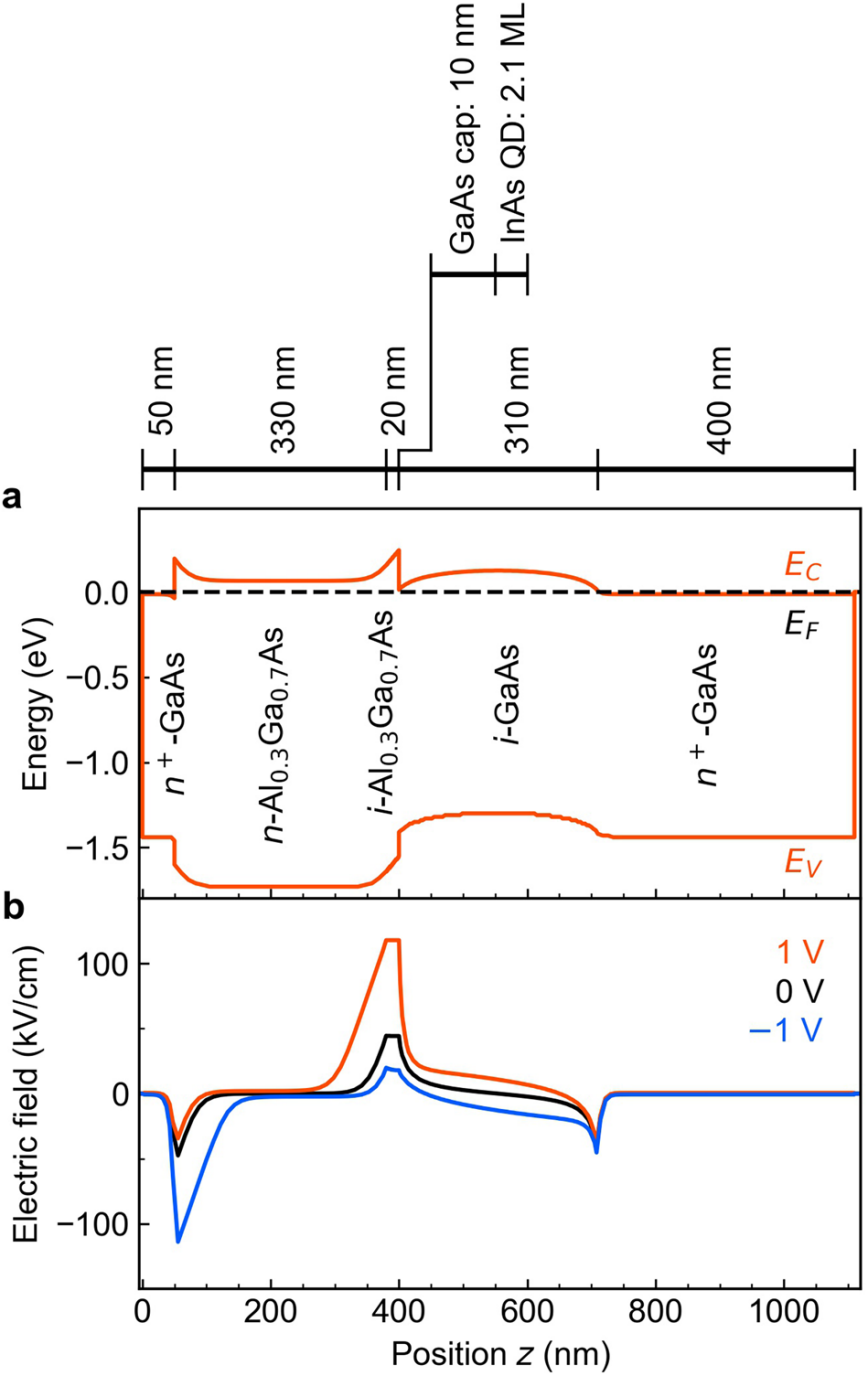
Structure of the device. (**a**) Calculation result for the band diagram of the device excluding the QD layer at 300 K using nextnano3. The broken line indicates the Fermi level of the electron. At the Al_0.3_Ga_0.7_As/GaAs heterointerface (*z* = 400 nm), the Fermi level of the electron lies near the conduction-band minimum, indicating that the electron density at the interface is high. The actual device used in the experiments contains an InAs/GaAs QD layer as shown in the explanation of the layer positions on the top. (**b**) Distributions of the electric field in the device for three different bias voltages. Near the heterointerface, the internal electric field is strong, and thus most of the hot electrons generated by IR absorption can cross the heterointerface and are injected into the Al_0.3_Ga_0.7_As layer.

The photodetector developed in the present work has an *n–i–n* structure. The top and the bottom layers are *n*-type Al_0.3_Ga_0.7_As and *n*-type GaAs, respectively. The intrinsic region comprises an Al_0.3_Ga_0.7_As/GaAs heterostructure and the InAs QDs as indicated in Fig. 1a (positions of the layers are shown on the top). An efficient intraband transition can be expected by inserting a QD layer just beneath the heterointerface with a very thin GaAs spacer layer of 10 nm^55–57^. The numerically calculated band diagram of the *n–i–n* photodetector at 300 K for a bias voltage of 0 V is shown in Fig. 1a. The electric field distributions in the device for bias voltages of 0 and $\pm$ 1 V are plotted in Fig. 1b. For these calculations, we ignored the InAs QDs to simplify the calculation and the bias was applied at the device surface. The Al_0.3_Ga_0.7_As barrier height at the conduction-band discontinuity is approximately 220 meV. The calculation predicts that the Fermi level of the electron at the heterointerface at *z* = 400 nm lies near the conduction-band edge of GaAs, i.e., electrons accumulate here and reach a relatively high density. We note that two heterointerfaces exist in this device: the *n*^+^-GaAs/*n*-Al_0.3_Ga_0.7_As near *z* = 50 nm, and the intrinsic Al_0.3_Ga_0.7_As/GaAs heterointerface at *z* = 400 nm. The latter is mainly discussed in the following, as the InAs QD and wetting layer states are located near this interface. Furthermore, the triangular potential wells at these two interfaces lead to the formation of confined states that also contribute to the measured IR photocurrent. When a positive bias is applied to the surface, the electrons near the heterointerface are excited by IR light and immediately afterwards these hot electrons drift towards the Al_0.3_Ga_0.7_As layer. The hot electrons can be efficiently injected into the Al_0.3_Ga_0.7_As layer, because the internal electric field near the heterointerface is strongly positive. On the other hand, when a negative bias is applied, a part of the hot electrons generated by IR absorption stays on the GaAs side.

**Figure 2:**
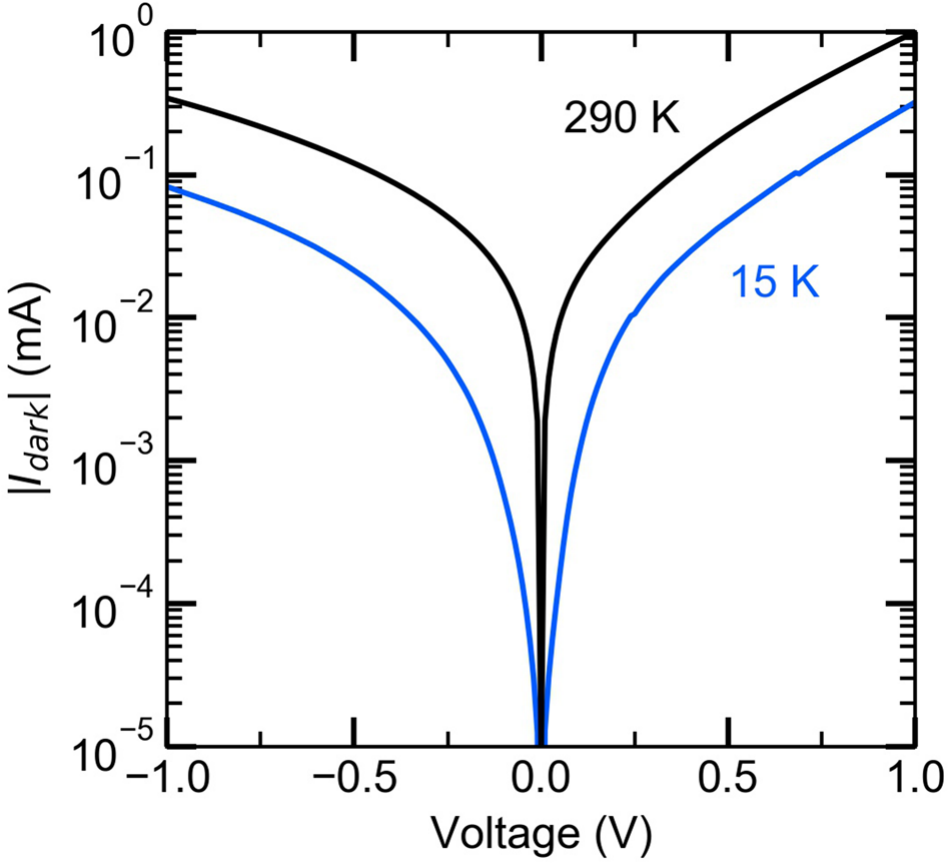
Current–voltage characteristics without light illumination. Bias voltage dependence of the absolute value of the dark current | (*I*_*dark*_) | at 15 and 290 K. The dark current at 290 K is approximately three times larger than that at 15 K.

**Figure 3:**
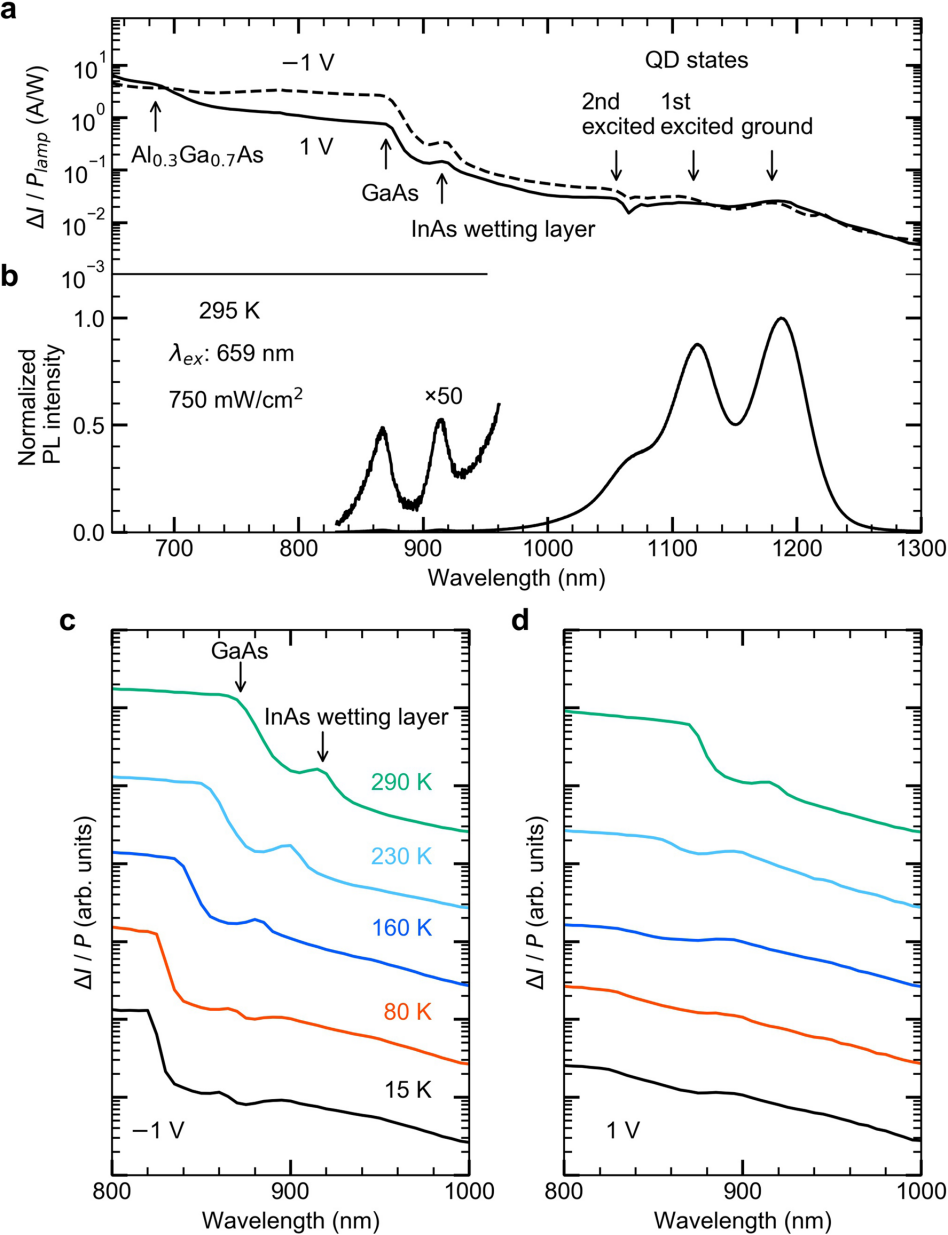
Spectral responses of the device. (**a**) Optical responsivity Δ*I/P* spectrum in the wavelength range from 650 to 1,300 nm at 295 K. (**b**) Normalized PL spectrum at 295 K. (**c**, **d**) Δ*I/P* spectra at various temperatures for bias voltages of ± 1 V, respectively.

Figure 2 plots the actually measured the absolute value of the dark current $\left|{I}_{dark}\right|$ as a function of the bias voltage for sample temperatures of 15 and 290 K. By applying either a positive or negative bias to the surface, electrons are extracted from the Al_0.3_Ga_0.7_As or GaAs sides and a current flow in opposite directions is established. The current–voltage (*I–V)* characteristics are asymmetric with respect to the sign of the bias voltage due to the asymmetry of the heterostructure. The ${I}_{dark}$ for negative bias voltages is smaller, because the current is influenced by the opposite electric field formed in the intrinsic GaAs layer. The enhancement in the dark current when the temperature changes from 15 to 290 K is not significantly large, which is due to the relatively high dark current at 15 K as compared with previously reported results in spite of the moderate dark current at 290 K (for example, see Ref.^38^). The high dark current observed at the low temperature is caused by the shallow Fermi level in the intrinsic layer. The shallow Fermi level is necessary to design the two-dimensional electron accumulation at the hetero-interface as indicated in Fig. 1 and also gives rise to a sensitive population even at the low temperature. Figure 3a shows the room-temperature responsivity spectrum, which is defined by the photocurrent $\Delta I$ (difference between total current $I$ and ${I}_{dark}$) normalized by the excitation light power *P*. We confirm an absorption edge at the GaAs band gap (near 875 nm), and for excitation wavelengths shorter than 875 nm, electrons are mainly excited via direct band-to-band transitions in GaAs. The Δ*I* for excitation wavelengths smaller than 685 nm (range for direct excitation of Al_0.3_Ga_0.7_As) is almost independent of the sign of the bias voltage. Conversely, obvious anisotropy appears for excitation wavelengths larger than 685 nm. The $\Delta I$ at positive bias is smaller, since the carrier transport towards the Al_0.3_Ga_0.7_As layer is strongly affected by the Al_0.3_Ga_0.7_As barrier, i.e., electrons need to cross the Al_0.3_Ga_0.7_As barrier. Below the GaAs band gap energy we confirmed several peaks. The peak at 915 nm corresponds to interband transitions in the InAs wetting layer. The QD ground state appears at 1,190 nm, and the 1st and 2nd QD excited states are observed at 1,120 and 1,060 nm, respectively. These interband transitions completely agree with the room-temperature photoluminescence (PL) signals, which are shown in Fig. 3b.

The influence of the asymmetric heterostructure on the photocurrent is investigated with the temperature dependence of $\Delta I$ in the wavelength region 800–1,000 nm. Figure 3c,d compare the results of the optical responses $\Delta I/P$ detected for bias voltages of ± 1 V, respectively. The absorption edges corresponding to GaAs and the InAs wetting layer are clearly observed. For − 1 V (see Fig. 3c), electrons are transported towards the bottom electrode. As the temperature decreases, the absorption edge of GaAs shifts to shorter wavelengths and becomes relatively steep due to exciton formation^59^. At lower temperatures, the signal induced by absorption via the InAs wetting layer states is less pronounced, which is attributed to increased state filling at low temperatures (the filled state suppresses interband absorption). Moreover, the energy separation between the GaAs edge and the wetting layer states gradually becomes small with decreasing temperature, suggesting the Burstein-Moss shift. In contrast to the data for − 1 V, the low-temperature spectra obtained for a bias of 1 V are significantly different (see Fig. 3d). Although similar absorption edges are clearly observed at 290 K, these signals disappear at low temperatures. This is caused by the asymmetric heterostructure; electrons that are directly excited in the intrinsic GaAs layer, are blocked by the Al_0.3_Ga_0.7_As barrier at the positive bias condition since thermal activation over the large barrier is hardly possible at low temperatures. Therefore, at low temperatures, excited electrons accumulate at the heterointerface. The photocurrent spectrum at 290 K indicates that thermal activation over the barrier at the heterointerface is not negligible. These results clearly demonstrate the effect of the Al_0.3_Ga_0.7_As barrier on the carrier transport in the *n–i–n* photodetector.

### Optical response in the IR wavelength region

**Figure 4:**
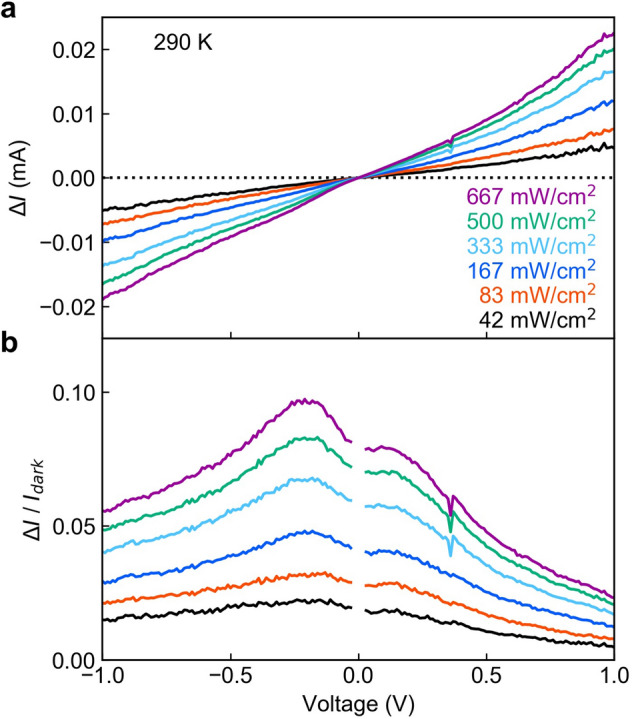
Voltage dependence of photocurrent under continuous illumination with IR light at 1,319 nm. (**a**) Data for Δ*I* and (**b**) Δ*I /I*_*dark*_ at 290 K for six different excitation power densities. Δ*I /I*_*dark*_ around 0 V is not shown in order to avoid division by zero. Δ*I/I*_*dark*_ exhibits two peaks at ± 0.2 V.

**Figure 5:**
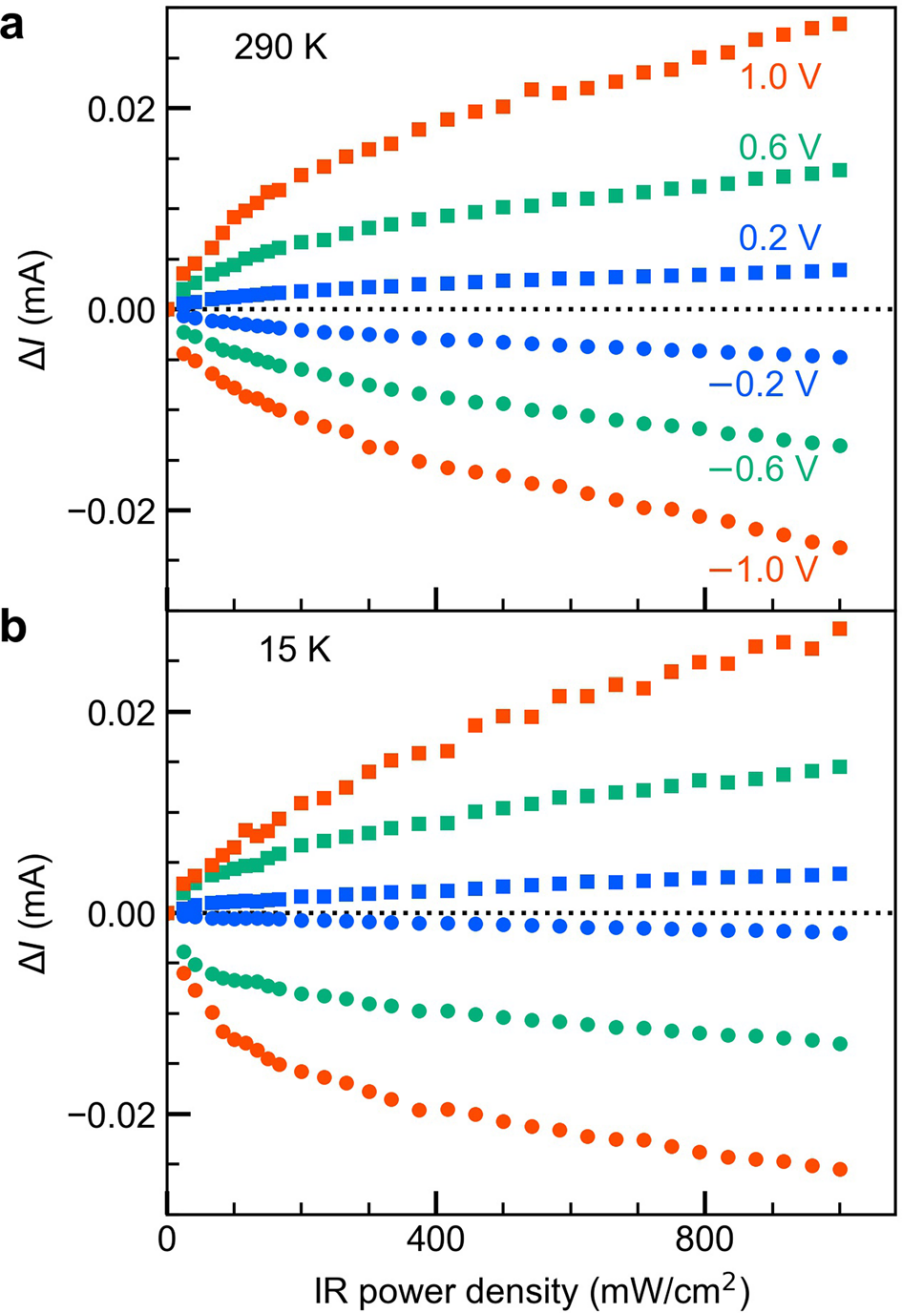
Dependence of ∆*I* on the IR power density at different bias voltages. (**a**) Data at room temperature (290 K) and (**b**) at 15 K. The photocurrent ∆*I* is almost independent of the temperature.

Next, we present the details of the IR response due to intraband transitions near the conduction-band discontinuity of the heterointerface. Figure 4a shows the room-temperature photocurrent that is measured upon excitation with IR light as a function of the bias voltage. The employed IR light source is a solid-state laser with a wavelength of 1,319 nm. The IR photon energy is thus smaller than the band gap energy of GaAs, i.e., the IR light cannot induce interband transitions and only causes intraband transitions near the heterointerface^55^. The IR laser spot area at the device surface was approximately 0.012 cm^2^. In Fig. 4a, the measured photocurrent $\Delta I$ exhibits a monotonic increase with the bias voltage, and the magnitude or the slope of the $\Delta I$–*V* curve increases with higher IR power density. To eliminate the influence of the dark-current structure, we analyzed $\Delta I/{I}_{dark}$ as a function of the bias voltage as shown in Fig. 4b. The resulting spectrum exhibits two peaks at about ± 0.2 V. We find that the peak near $-$ 0.2 V is dominant, indicating that the detection efficiency reaches its maximum near $-$ 0.2 V. This is a result of the asymmetry of the dark current shown in Fig. 2. The electron density at the heterointerface decreases due to the electric-field-induced carrier extraction, which weakens the intraband absorption. This trade-off relationship governs the voltage dependence of $\Delta I/{I}_{dark}$ in Fig. 4b. Figure 5a,b summarize the dependences of $\Delta I$ on the IR power density for high (290 K) and low temperatures (15 K), respectively. As expected from the results in Fig. 4a, $\Delta I$ exhibits a gradual increase with the IR power density and behaves almost symmetric with respect to the polarity of the bias voltage. $\Delta I$ shows a sublinear increase with the IR power density, which arises from lowering the quasi-Fermi level due to the strong intraband excitation. While ${I}_{dark}$ at 290 K is approximately three times larger than that at 15 K (Fig. 2), $\Delta I$ is almost independent of the device temperature. This evidences that the detected photocurrent is hardly influenced by thermal artifacts.

**Figure 6:**
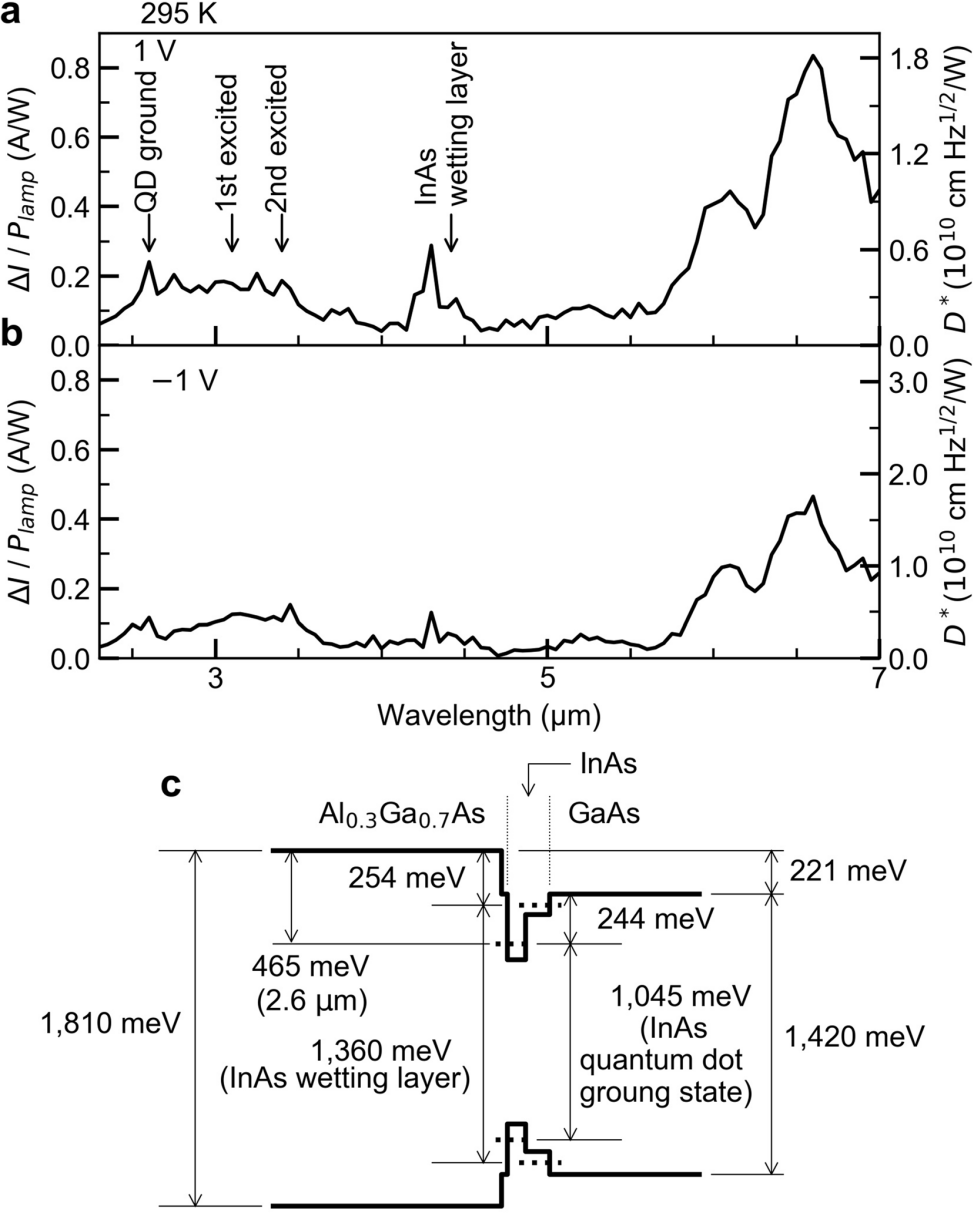
IR photodetector device performance. (**a**) Optical responsivity Δ*I/P*_lamp_ and detectivity D* spectra in the MWIR region at 295 K for bias voltages 1 V and (**b**) − 1 V. (**c**) Energy states of the confined electrons near the heterointerface at about 300 K. (Figure reproduced from Asahi et al. ^55^. Copyright 2017 The authors under the Creative Commons Attribution 4.0).

Finally, we discuss the spectra of the room-temperature optical responsivity $\Delta I/P$ of this device in the MWIR region from 2.3 to 7.0 µm. Figure 6a,b show the spectra obtained for 1 and − 1 V at which the responsivity becomes maximum in this experiment, respectively. Several peaks corresponding to the intraband transitions near the heterostructure appear in the spectra. We note that strong responsivity signals are observed in the MWIR region even at 295 K (that is, room temperature). It is noted that the responsivity in the wavelength region smaller than 1.2 µm, where the interband transitions of QDs dominantly occur, is strongly reduced. $\Delta I/P$ in this wavelength region includes enhanced photocurrent caused by increased thermal escape of electrons additionally generated by the interband excitation. The energy states near the heterointerface at about 300 K are illustrated in Fig. 6c. The numerical values indicated in this figure are the same as those previously reported for the same intrinsic heterostructure design^55^. The values were determined by the responsivity and PL spectra and the energy differences in the conduction-band lineup were evaluated by the temperature dependences of $\Delta I$ and the PL intensity. The resulting possible intraband transitions are indicated in this band diagram.

Firstly, we discuss the result obtained for a bias of 1 V (Fig. 6a), where the excited electrons drift towards the front and are transported across the Al_0.3_Ga_0.7_As layer. The transition from the QD ground state to the conduction-band edge of Al_0.3_Ga_0.7_As appear at ~ 2.6 µm, and signals of the transitions from the QD excited states lie at ~ 3.1 µm and ~ 3.6 µm. The signal at ~ 4.3 µm is attributed to the transition from the InAs wetting layer state to the conduction-band edge of Al_0.3_Ga_0.7_As. A weak and broad signal appears at ~ 5.3 µm, which is close to the conduction-band discontinuity of 221 meV at the Al_0.3_Ga_0.7_As/GaAs heterointerface. We also confirm a strong peak at ~ 6.6 µm (the dip structure at ~ 6.3 µm is due to the absorption by H_2_O). The maximum responsivity is 0.83 A/W at ~ 6.6 µm. This value is almost the same as the interband transition in bulk and constitutes the largest responsivity so far^3,19,20,26,31,38,39^. The signal at ~ 6.6 µm is attributed to a transition from a state located above the GaAs conduction-band edge to the edge of Al_0.3_Ga_0.7_As conduction-band. A possible state contributing to this transition is the quantized state formed in the triangular potential well at the heterointerface. According to the simulation at a bias of 1 V, a quantized state is formed at 185 meV below the Al_0.3_Ga_0.7_As conduction-band edge. This energy difference is in fair agreement with the signal at ~ 6.6 µm. Since the Fermi energy is 186 meV below the conduction-band edge of Al_0.3_Ga_0.7_As, the quantized state formed in the triangular potential well is occupied by electrons and, therefore, a strong intraband transition is observed upon illumination with IR light. Here, InAs QDs play a key role even for the significant detection strength for the AlGaAs/GaAs heterointerface. Generally, the optical selection rule of the intraband transition of electrons in an ideal two-dimensional structure is forbidden for light irradiating the two-dimensional plane perpendicularly^60^. The finite thickness of the accumulation layer relaxes the selection rule, and, moreover, InAs QDs play a role enhancing the intraband transition probability. It is well known that the electronic wavefunctions in QDs are quantized on all three dimensions, and light of all polarization directions induces intraband transitions^61^. Thus, the presence of QDs near the heterointerface dramatically improves the intraband transition probability at the heterointerface.

The responsivity spectrum obtained at − 1 V is provided in Fig. 6b. Under this bias condition, the electrons excited by the IR photons are transported towards the *n*-type GaAs layer. A potential barrier for the carrier transport exists in the intrinsic GaAs layer as shown in Fig. 1a. The estimated height of this barrier is 26 meV at − 1 V. The transition energy of weak, broad signals appearing around 3.2 µm is approximately 382 meV which is larger than the energy difference between the QD ground state and the top of the potential barrier formed in GaAs. This means that the final state of the intraband transition exists 138 meV above the GaAs edge at the heterointerface. The strongest signal at − 1 V is observed at ~ 6.6 µm. This can be attributed to the GaAs/Al_0.3_Ga_0.7_As heterointerface near the front surface of the device (located 50 nm below the surface). From the theoretical simulation of the band profile at a bias of − 1 V, we find that a quantized state in the triangular potential well formed near this heterointerface is formed (212 meV below the Al_0.3_Ga_0.7_As conduction-band minimum). For a bias of − 1 V, the Fermi level is 200 meV below the conduction-band edge of Al_0.3_Ga_0.7_As, and thus this quantized state is occupied. The energy difference of 212 meV corresponds to ~ 5.8 µm, which is shorter than the observed peak position. The shift of the predicted intraband absorption peak towards longer wavelengths is explained with tunneling. Since the *n*^+^-GaAs/*n*- Al_0.3_Ga_0.7_As heterointerface does not contain an intrinsic layer, the potential barrier formed in *n*-Al_0.3_Ga_0.7_As becomes very thin and tunneling across the barrier lowers the effective barrier height. The responsivity at ~ 6.6 µm under the bias of − 1 V in Fig. 6b is weaker than that at 1 V in Fig. 6a, suggesting that the presence of the QDs increases the strength of the intraband transition near the heterointerface.

The scale for the specific detectivity ${D}^{*}$ is provided on the right-hand sides of Fig. 6a,b. The detectivity of a photodetector can be defined as the ratio of signal to noise, and is given by ${D}^{*}=R\sqrt{A\Delta f}/{i}_{N}$, where $R$ is the responsivity, $A$ the detector area, $\Delta f$ the noise bandwidth, and ${i}_{N}$ is the dark-current noise (square root of the sum of squares of Johnson noise $\sqrt{4{k}_{B}T/r} (A)$ and Shot noise $\sqrt{2q{I}_{dark}}$ (A). In the expression of ${i}_{N}$, ${k}_{B}$ is the Boltzmann constant, $T$ the temperature, $r$ the differential resistance at each bias, and $q$ is the elementary charge. Here, $\Delta f=1$ Hz. The noise we took into account is a white noise which is independent of the frequency, and, therefore, the estimated ${D}^{*}$ is the value not affected by the $1/f$ noise. The ${D}^{*}$ spectrum is proportional to the responsivity, and the maximum value is approximately $1.8\times {10}^{10}$ cmHz^1/2^/W at ~ 6.6 µm for the bias voltage of 1 V. This value is comparable to the ${D}^{*}$ of current photodetectors operating at low temperatures^62^. In addition, we calculated black-body ${D}^{*}(500 K)$ using the ${D}^{*}$ spectra shown in Fig. 6. The calculated ${D}^{*}(500 K)$ is 2.0 × 10^9^ at 1 V, which is a promising value as compared with the previously reported values for mid-IR sensors operable at room temperature^62^.

In conclusion, we proposed a new structure for an IR photodetector and demonstrated operation at room temperature. This device is sensitized by InAs QDs embedded near an Al_0.3_Ga_0.7_As/GaAs heterointerface and electrons that have been accumulated at the heterointerface are transferred to the Al_0.3_Ga_0.7_As barrier by absorbing IR photons and following drift due to the electric field at the interface. We systematically investigated the photoelectric conversion properties and measured the MWIR responsivity. The heterostructure causes a carrier transport that is asymmetric with respect to the polarity of the applied bias. The responsivity spectrum measured at room temperature shows several peaks in the MWIR wavelength region corresponding to transitions from the InAs/GaAs QD and wetting layer states as well as the quantized state of the triangular potential well at the two-dimensional heterointerface. The responsivity is almost independent of the temperature and the maximum value at 295 K is 0.8 A/W at ~ 6.6 µm for a bias of 1 V, where the specific detectivity is $1.8\times {10}^{10}$ cmHz^1/2^/W. These results suggest that the proposed heterostructure including a QD layer is promising for IR photodetectors that provide a high detectivity even at room temperature.

## Methods

### Device simulation

We employed nextnano3^63,64^ to calculate the band diagram of our device for different bias voltages.

### Device fabrication

The device was fabricated on a *n*^+^-GaAs (001) substrate using solid-source molecular beam epitaxy. The substrate temperature during the growth was monitored using an IR pyrometer. The beam-equivalent pressure of the As_2_ flux was 1.15 × 10^−3^ Pa. The device structure is shown in Fig. 1a. First, a 400-nm-thick *n*^+^-GaAs (Si: 2.5 × 10^18^ cm^−3^) buffer layer was grown at 550 °C. This was followed by growth of an intrinsic GaAs layer (300 nm), the InAs QDs, a GaAs capping layer (10 nm), and an Al_0.3_Ga_0.7_As layer (20 nm). The thickness equivalent to the deposited amount of InAs was 0.64 nm (2.1 monolayers). The GaAs layer below the InAs QD layer was grown at 550 °C. The InAs QDs and the subsequent 10-nm-thick GaAs capping layer were grown at 490 °C. Then, 20-nm-thick *i-*Al_0.3_Ga_0.7_As and 330-nm-thick *n-*Al_0.3_Ga_0.7_As (Si: 1.0 × 10^17^ cm^−3^) was grown at 500 °C. Finally, a 50-nm-thick *n*^+^-GaAs (Si: 2.5 × 10^18^ cm^−3^) layer was deposited as contact layer. Ohmic Au/Au–Ge contacts were deposited on the top and bottom surfaces. Here, the top electrode has a comb-type pattern with 0.25-mm-thick grid fingers and 0.45-mm spacing between them. The dimensions of the final device were 4 mm × 4 mm.

### Current–voltage (*I–V)* measurement

We measured the dark current and photocurrent at various bias voltages by using a sourcemeter (Keithley 2400). The IR light source used to induce the intraband transitions was a solid-state laser with a wavelength of 1,319 nm. The laser spot area at the device surface was approximately 0.012 cm^2^. A variable neutral density filter was used to control the excitation power density.

### Photoluminescence (PL) measurement

The PL measurements were performed using a continuous-wave laser diode with a wavelength of 659 nm. The excitation power density was 750 mW/cm^2^. The PL signal was dispersed by using a 30-cm single monochromator and detected by an InGaAs diode array.

### Optical responsivity $\Delta I/P$ measurement

For excitation in the 0.65–1.3 µm region, we used a supercontinuum white laser dispersed by a 270-mm single monochromator. The spot area of the excitation light was approximately 0.03 cm^2^. The power density region was 13–50 mW/cm^2^ in each wavelength. A thermal power sensor (THORLABS S401C) was used to measure the light intensity. For the excitation in the MWIR region, we employed a SiC lamp dispersed by a 320-mm single monochromator. The excitation light was focused on the total area of the device. The power density region was 58–370 µW/cm^2^ in each wavelength. We measured the photocurrent at various bias voltages by using a sourcemeter (Keithley 2400).
